# Symbiosis between *Candidatus* Patescibacteria and Archaea Discovered in Wastewater-Treating Bioreactors

**DOI:** 10.1128/mbio.01711-22

**Published:** 2022-08-31

**Authors:** Kyohei Kuroda, Kyosuke Yamamoto, Ryosuke Nakai, Yuga Hirakata, Kengo Kubota, Masaru K. Nobu, Takashi Narihiro

**Affiliations:** a Bioproduction Research Institute, National Institute of Advanced Industrial Science and Technology (AIST), Toyohira‐ku, Sapporo, Hokkaido, Japan; b Bioproduction Research Institute, National Institute of Advanced Industrial Science and Technology (AIST), Tsukuba, Ibaraki, Japan; c Department of Frontier Sciences for Advanced Environment, Graduate School of Environmental Studies, Tohoku University, Aramaki, Aoba-ku, Sendai, Miyagi, Japan; Oregon State University

**Keywords:** Candidate Phyla Radiation (CPR), *Candidatus* Patescibacteria, Archaea, *Candidatus* Yanofskybacteria/UBA5738, symbiosis, fluorescence *in situ* hybridization (FISH), transmission electron microscopy (TEM), shotgun metagenomic analysis

## Abstract

Each prokaryotic domain, Bacteria and Archaea, contains a large and diverse group of organisms characterized by their ultrasmall cell size and symbiotic lifestyles (potentially commensal, mutualistic, and parasitic relationships), namely, *Candidatus* Patescibacteria (also known as the Candidate Phyla Radiation/CPR superphylum) and DPANN archaea, respectively. Cultivation-based approaches have revealed that *Ca*. Patescibacteria and DPANN symbiotically interact with bacterial and archaeal partners and hosts, respectively, but that cross-domain symbiosis and parasitism have never been observed. By amending wastewater treatment sludge samples with methanogenic archaea, we observed increased abundances of *Ca*. Patescibacteria (*Ca*. Yanofskybacteria/UBA5738) and, using fluorescence *in situ* hybridization (FISH), discovered that nearly all of the *Ca.* Yanofskybacteria/UBA5738 cells were attached to *Methanothrix* (95.7 ± 2.1%) and that none of the cells were attached to other lineages, implying high host dependency and specificity. *Methanothrix* filaments (multicellular) with *Ca.* Yanofskybacteria/UBA5738 attached had significantly more cells with no or low detectable ribosomal activity (based on FISH fluorescence) and often showed deformations at the sites of attachment (based on transmission electron microscopy), suggesting that the interaction is parasitic. Metagenome-assisted metabolic reconstruction showed that *Ca.* Yanofskybacteria/UBA5738 lacks most of the biosynthetic pathways necessary for cell growth and universally conserves three unique gene arrays that contain multiple genes with signal peptides in the metagenome-assembled genomes of the *Ca.* Yanofskybacteria/UBA5738 lineage. The results shed light on a novel cross-domain symbiosis and inspire potential strategies for culturing CPR and DPANN.

## OBSERVATION

One major lineage of the domain Bacteria, *Candidatus* Patescibacteria (also known as the Candidate Phyla Radiation/CPR superphylum) (1, 2), is a highly diverse group of bacteria that widely inhabits natural (3, 4) and artificial ecosystems (5–7) and is characterized by its small cell and genome sizes (8) and by its poor (genomically predicted) abilities to synthesize cellular building blocks (4), which is suggestive of a symbiotic dependency for cell growth. Most members remain uncultured, leaving major knowledge gaps in the range and nature of their symbioses (i.e., commensal, mutualistic, and parasitic relationships) (3), although a few cultivation-based and microscopy-based studies have demonstrated host-specific symbiotic and parasitic interactions between *Ca*. Patescibacteria and other bacteria; e.g., *Ca*. Saccharimonadia with Actinomycetota (formerly known as Actinobacteria) (9) and *Ca*. Gracilibacteria with Gammaproteobacteria (10, 11). This suggested a specialization toward bacteria-bacteria symbioses, especially given the parallels with DPANN, an archaeal analog which has only been observed to interact with other archaea (4, 12); however, one study has reported symbiosis between *Ca*. Patescibacteria and eukarya (13), indicating that the *Ca*. Patescibacteria host range reaches beyond bacteria. Here, based on our previous observations of predominant *Ca*. Patescibacteria members and methanogenic archaea (“methanogens”) (6, 7), we hypothesize that some *Ca*. Patescibacteria may symbiotically interact with archaea and use exogenous archaea to culture potential archaea-dependent *Ca*. Patescibacteria.

To create conditions conducive to the growth of archaea-dependent *Ca*. Patescibacteria, we took a strategy similar to that of virus or phage cultivation, in which exogenous methanogenic archaea were grown in the presence of *Ca*. Patescibacteria that would presumably grow using molecules derived from these active hosts (i.e., through symbiosis and parasitism). We chose acetate-utilizing methanogens as the partners, as they ubiquitously inhabit methanogenic ecosystems (5, 6, 14), form symbiotic interactions with bacteria (14), utilize an energy source (acetate) that is generally noninhibitory to organotrophs (unlike other methanogen substrates, such as H_2_ or formate [15]), and conveniently have a highly distinguishable cell morphology/structure that is easily differentiable from those of other organisms (i.e., easily traceable under a microscope). To culture *Ca*. Patescibacteria that may interact with archaea, we used microbial community samples from a bioreactor (“sludge”) that was particularly abundant in *Ca*. Patescibacteria (7) as starting material and amended them with acetate-utilizing methanogens (Methanothrix soehngenii GP6 and Methanosarcina barkeri MS) as symbiotic partners and acetate as an energy source for the archaea (Text S1). Potential growth factors (yeast extract, various amino acids, and nucleoside monophosphates) were also provided, as *Ca*. Patescibacteria are known to have poor biosynthetic capacities (4).

10.1128/mbio.01711-22.1 TEXT S1 The file containing materials and methods. Download Text S1, PDF file, 0.2 MB. Copyright © 2022 Kuroda et al. 2022 Kuroda et al. https://creativecommons.org/licenses/by/4.0/ This content is distributed under the terms of the Creative Commons Attribution 4.0 International license.

**FIG 1:**
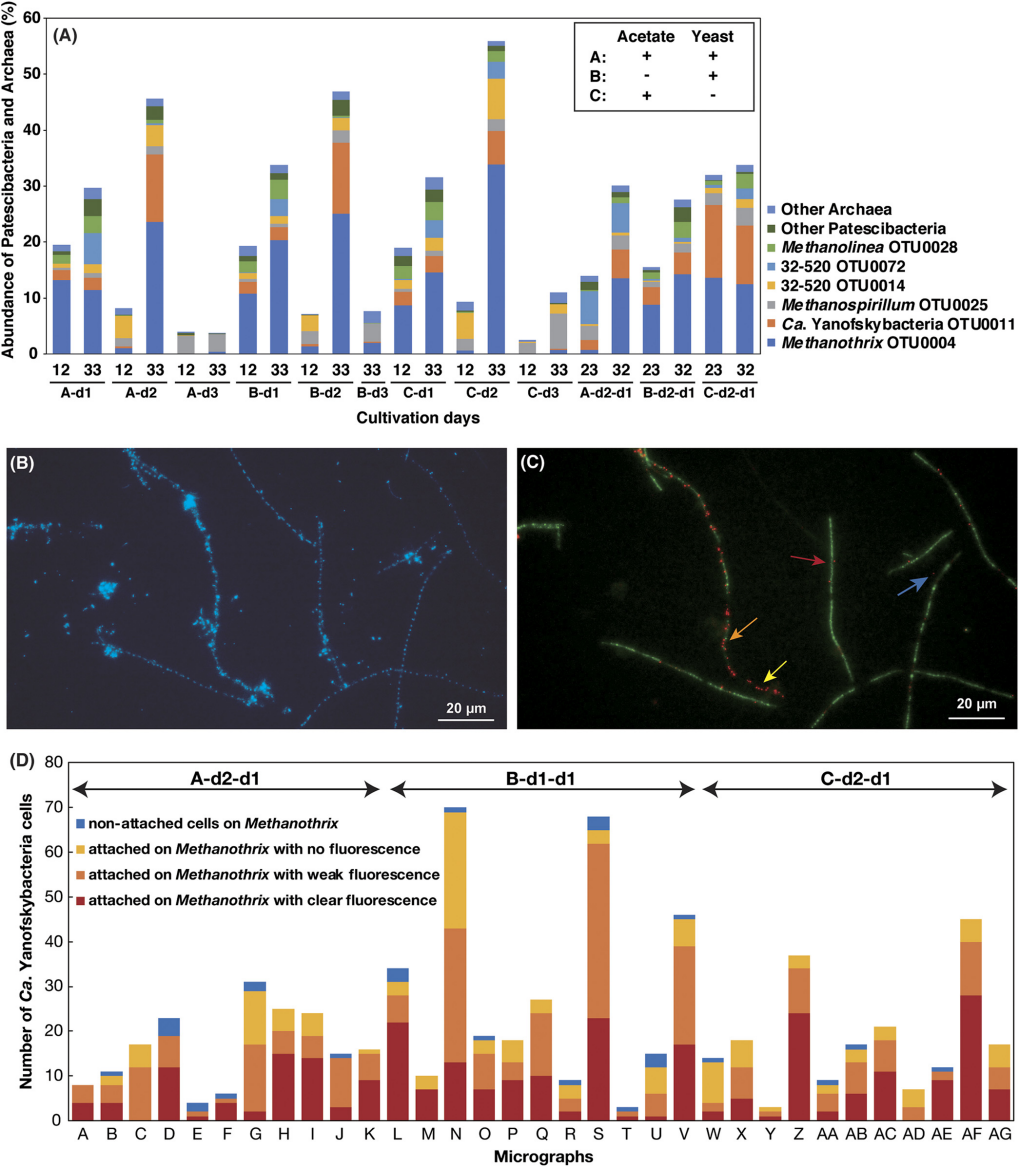
(A) Relative abundance of predominant *Candidatus* Patescibacteria and Archaea in the culture systems based on 16S rRNA gene sequence analysis. Micrographs of (B) 4′,6-diamidino-2-phenylindole dihydrochloride staining and (C) fluorescence *in situ* hybridization (FISH) obtained from culture system B-d1-d1 on day 23 as well as the (D) counted numbers of *Ca*. Yanofskybacteria cells in 33 micrographs. (C) The microorganisms in the panel were labeled with *Ca.* Yanofskybacteria-targeting Pac_683-Cy3 probe (red) and *Methanothrix*-targeting MX825-FITC probe (green). Blue, yellow, orange, and red arrows in (C) indicate nonattached *Ca.* Yanofskybacteria cells on *Methanothrix*, attached on *Methanothrix* with no fluorescence, attached on *Methanothrix* with weak fluorescence, and attached on *Methanothrix* with clear fluorescence, respectively, and these colors are consistent with those in the bar diagrams of (D). Culture systems were amended with (A) acetate and yeast extract, (B) yeast extract, and (C) acetate for carbon sources. A-d2-d1, B-d1-d1, and C-d2-d1 were subcultures transferred from A-d2, B-d1, and C-d2, respectively.

In the cultivation experiments, we performed serial dilutions (10^−1^, 10^−3^, 10^−4^, and 10^−6^, defined as d1–d4) of the sludge-methanogen mixture to help eliminate low-abundance bacteria that may have interfered with the *Ca*. Patescibacteria growth. In some cultures with confirmed gas production, we detected the enrichment (increased relative abundances up to 12.1% in A-d2 on day 33) of a population of an uncultured clade of *Ca*. Patescibacteria, namely, *Ca*. Yanofskybacteria OTU0011, which belongs to class *Ca*. Paceibacteria (formerly known as Parcubacteria/OD1 [1]) (Fig. 1A), and we also observed many small cells (<1 μm in diameter) that were consistently attached to cells with a morphology characteristic of *Methanothrix* (long rods of approximately 0.8 μm in diameter with blunt ends strung together, forming multicellular filaments), with the number of attached cells increasing as the culture aged (Fig. S1A and S1B). To further eliminate other nontarget populations in the culture (i.e., “enrich” the target organisms), we subcultured those abundant in small cells and microscopically confirmed the continued physical attachment of small cells with *Methanothrix*-like cells (Fig. S1C and S1D). Three independent subcultures (defined as A-d2-d1, B-d1-d1, and C-d2-d1) retaining *Ca.* Yanofskybacteria (each amended with acetate and/or yeast extract) with high abundance (1.7 to 13.1%) (Fig. 1A) were used for further microscopy observation.

10.1128/mbio.01711-22.2 FIG S1 Phase-contrast micrographs of (A–D) the small cells attached on *Methanothrix*-like cells in culture systems A-d2 on day 33 (A and B) and C-d2-d1 on day 23 (C and D). Yellow squares indicate high magnification parts of (A) and (B). White arrows indicate the colorless cells of the *Methanothrix*-like structure. Download FIG S1, JPG file, 1.2 MB. Copyright © 2022 Kuroda et al. 2022 Kuroda et al. https://creativecommons.org/licenses/by/4.0/ This content is distributed under the terms of the Creative Commons Attribution 4.0 International license.

Through fluorescence *in situ* hybridization (FISH), which allowed for the differentiation of target populations with fluorescence microscopy, we successfully verified that the small cells attached to the surfaces of the *Methanothrix* filaments were indeed *Ca.* Yanofskybacteria (with probes MX825 and Pac_683, respectively) (Fig. 1B and C; also see Fig. S2–S4). Across all subcultures, most *Methanothrix* filaments (59.3 ± 5.3%) were physically associated with *Ca*. Yanofskybacteria, and, more importantly, nearly all of the *Ca*. Yanofskybacteria cells (95.7 ± 2.1%) were attached to *Methanothrix* filaments, with none attached to other hosts and only a small fraction remaining (4.2 ± 2.1%) unattached, showing that the physical attachment and symbiosis are *Methanothrix*-specific (Fig. 1D).

10.1128/mbio.01711-22.3 FIG S2 Micrographs of (A and E) phase-contrast, (B and F) 4′,6-diamidino-2-phenylindole dihydrochloride staining, and (C, D, G, and F) fluorescence *in situ* hybridization (FISH) obtained from culture system A-d2-d1 on day 23. (C and G) The *Ca.* Yanofskybacteria-targeting Pac_683-Cy3 probe and (D and H) the *Methanothrix*-targeting MX825-FITC probe. Blue, yellow, orange, and red arrows indicate nonattached *Ca.* Yanofskybacteria cells on *Methanothrix*, attached on *Methanothrix* with no fluorescence, attached on *Methanothrix* with weak fluorescence, and attached on *Methanothrix* with clear fluorescence, respectively, and these colors are consistent with those in the bar diagrams in Fig. 1D. Download FIG S2, JPG file, 1.7 MB. Copyright © 2022 Kuroda et al. 2022 Kuroda et al. https://creativecommons.org/licenses/by/4.0/ This content is distributed under the terms of the Creative Commons Attribution 4.0 International license.

**FIG 2:**
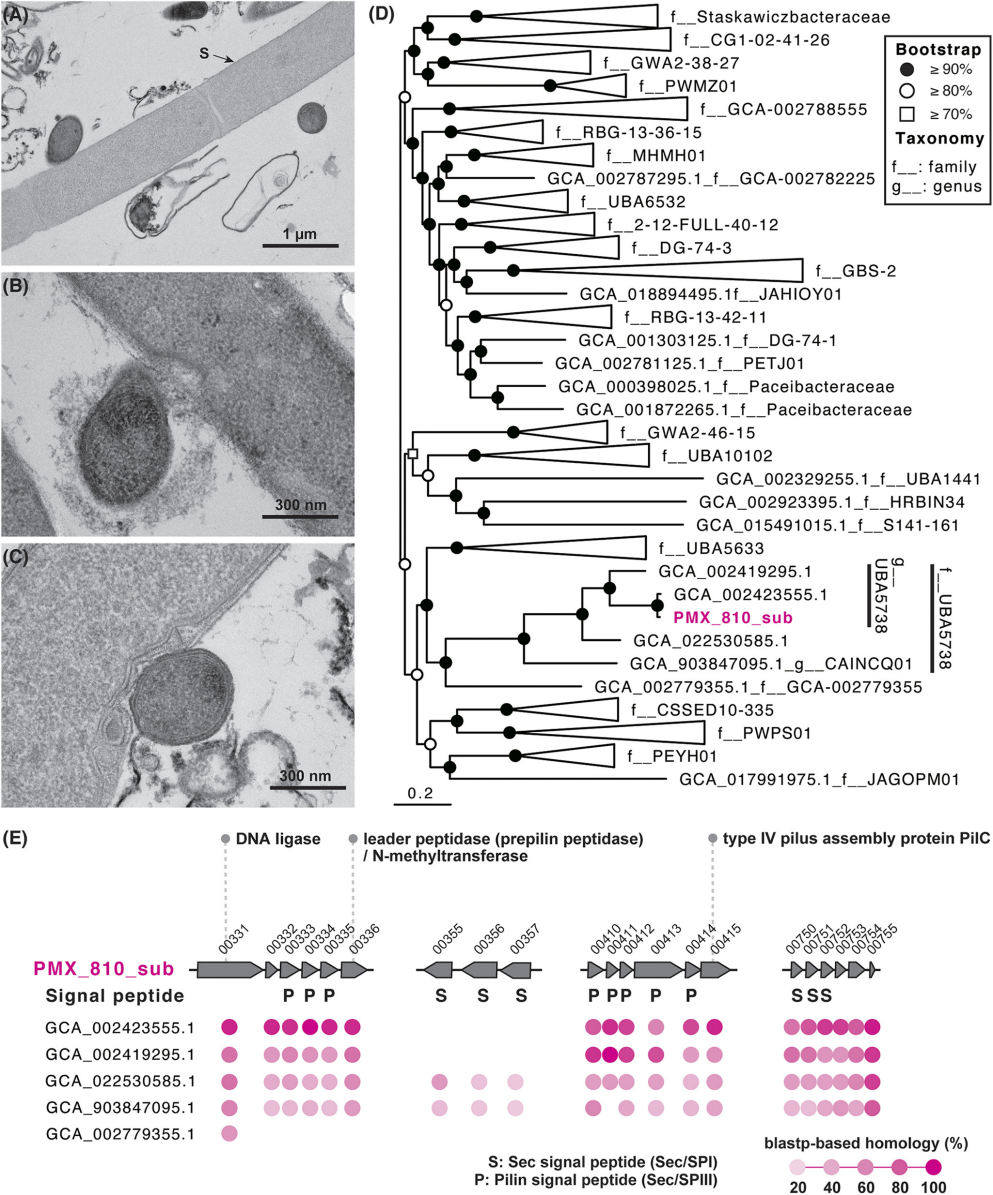
(A–C) Transmission electron micrographs of small coccoid-like submicron cells attached on the *Methanothrix*-like cells in culture system A-d2 on day 40. S indicates sheath structures of the *Methanothrix*-like cells. (D) Phylogenetic tree of order *Ca*. Paceibacterales based on concatenated phylogenetic marker genes of GTDBtk 2.0.0 (ver. r207). The phylogenetic position of the metagenomic bin PMX_810_sub is shown in pink color. (E) Gene arrays containing multiple genes with signal peptides in *Ca.* Yanofskybacteria/UBA5738 and the family level uncultured lineage GCA-002779355. P and S indicate the sec signal peptide and the pilin signal peptide, respectively. The pink colored circles indicate a BLASTP-based homology (threshold of ≤ 1e−10) with metagenomic bin PMX_810_sub. No annotated genes are hypothetical proteins (based on the annotation using BlastKOALA in Table S3). Abbreviated locus tags are shown in (E) (e.g., “PMX_810_sub_00331” as “00331” in the row of PMX_810_sub).

Compared to the *Methanothrix* filaments that were free of ectosymbionts in the cultures, those physically associated with more than 5 *Ca.* Yanofskybacteria cells contained significantly larger areas (*P* < 0.05 for all cultures) with low ribosomal activity (based on FISH fluorescence) (Fig. S5). Moreover, a major fraction of the *Ca.* Yanofskybacteria cells (18.8 ± 1.9%) were associated with these low-activity *Methanothrix* cells. Though highly qualitative, many of the *Ca*. Yanofskybacteria cells (39.6 ± 6.1%) were attached to *Methanothrix* cells with weak fluorescence (e.g., Fig. S2H, S3H, and S4H), which may reflect the negative influence of attachment. Using transmission electron microscopy (TEM), we further observed that *Methanothrix* (sheathed filamentous cells) (16, 17) often had deformed cell walls where submicron coccoid-like cells (presumably *Ca.* Yanofskybacteria; 0.46 ± 0.13 μm long and 0.36 ± 0.07 μm wide, 0.0377 ± 0.0200 μm^3^ calculated cell volumes) were attached (Fig. 2A–C). The clearly negative influence of *Ca.* Yanofskybacteria implies that the symbiosis between *Ca.* Yanofskybacteria and *Methanothrix* is parasitic.

10.1128/mbio.01711-22.4 FIG S3 Micrographs of (A and E) phase-contrast, (B and F) 4′,6-diamidino-2-phenylindole dihydrochloride staining, (C, D, G, and F) fluorescence *in situ* hybridization (FISH) obtained from culture system B-d1-d1 on day 23. (C and G) The *Ca.* Yanofskybacteria-targeting Pac_683-Cy3 probe and (D and H) the *Methanothrix*-targeting MX825-FITC probe. Blue, yellow, orange, and red arrows indicate nonattached *Ca.* Yanofskybacteria cells on *Methanothrix*, cells attached on *Methanothrix* with no fluorescence, cells attached on *Methanothrix* with weak fluorescence, and cells attached on *Methanothrix* with clear fluorescence, respectively, and these colors are consistent with those in the bar diagrams in Fig. 1D. Download FIG S3, JPG file, 2.0 MB. Copyright © 2022 Kuroda et al. 2022 Kuroda et al. https://creativecommons.org/licenses/by/4.0/ This content is distributed under the terms of the Creative Commons Attribution 4.0 International license.

10.1128/mbio.01711-22.5 FIG S4 Micrographs of (A and E) phase-contrast, (B and F) 4′,6-diamidino-2-phenylindole dihydrochloride staining, (C, D, G, and F) fluorescence *in situ* hybridization (FISH) obtained from culture system C-d2-d1 on day 23. (C and G) The *Ca.* Yanofskybacteria-targeting Pac_683-Cy3 probe and (D and H) the *Methanothrix*-targeting MX825-FITC probe. White arrows indicate *Methanothrix*-cells without *Ca.* Yanofskybacteria cells. Blue, yellow, orange, and red arrows indicate nonattached *Ca.* Yanofskybacteria cells on *Methanothrix*, cells attached on *Methanothrix* with no fluorescence, cells attached on *Methanothrix* with weak fluorescence, and cells attached on *Methanothrix* with clear fluorescence, respectively, and these colors are consistent with those in the bar diagrams in Fig. 1D. Download FIG S4, JPG file, 1.9 MB. Copyright © 2022 Kuroda et al. 2022 Kuroda et al. https://creativecommons.org/licenses/by/4.0/ This content is distributed under the terms of the Creative Commons Attribution 4.0 International license.

10.1128/mbio.01711-22.6 FIG S5 Cell length proportions of clear fluorescence of *Methanothrix* filamentous cells calculated based on fluorescence *in situ* hybridization (FISH) signals using the *Methanothrix*-targeting MX825-FITC probe and the *Ca.* Yanofskybacteria-targeting Pac_683-Cy3 probe. The *Methanothrix* cells attached with >5 *Ca.* Yanofskybacteria cells were chosen for calculation. The statistical analysis was performed using Welch’s *t* test. Download FIG S5, JPG file, 1.2 MB. Copyright © 2022 Kuroda et al. 2022 Kuroda et al. https://creativecommons.org/licenses/by/4.0/ This content is distributed under the terms of the Creative Commons Attribution 4.0 International license.

Through shotgun metagenomic analysis, we successfully recovered a metagenome-assembled genome of *Ca.* Yanofskybacteria (PMX_810_sub; 0.8 Mb in total) and nearly full-length 16S rRNA gene sequences (Fig. 2D; Fig. S6; Tables S1 and S2). Based on 43 marker genes for *Ca*. Patescibacteria (1), the completeness and contamination of PMX_810_sub were estimated to be 90.7% and 0%, respectively. Phylogenetic classification based on SILVA v138.1 and GTDB r207 taxonomy confirmed the classification of the *Ca.* Yanofskybacteria (99.7% similarity with FPLM01004990) (Fig. S6; Table S2) and the *Ca*. Patescibacteria family UBA5738 of the order Paceibacterales (Fig. 2D; Table S1). The metagenome-assembled genome PMX_810_sub lacks many biosynthetic pathways (e.g., those for the biosynthesis of amino acids and fatty acids) (Table S3), suggestive of a host-dependent or partner-dependent lifestyle, as was also observed for other *Ca*. Patescibacteria members (4). Previously, gene arrays that contain small signal peptides have been found in pathogenic bacterial genomes and in *Ca*. Patescibacteria (18), which may be related to their parasitic potential (19). Indeed, all of the metagenome-assembled genomes that were affiliated with *Ca.* Yanofskybacteria/UBA5738 contained genes for protein secretion systems (e.g., SecADEFGY) (Table S3) and universally conserved three unique gene arrays that contained multiple genes with signal peptides that could be recognized by the aforementioned systems (and one additional gene array conserved in the two deep-branching genomes) (Fig. 2D and E). Interestingly, all of the genes included in these arrays encode hypothetical proteins, suggesting that, if these genes are involved in interactions with the host, the mechanism is unrelated to known forms of parasitism. Further investigation using transcriptomics and proteomics is necessary to clarify the mechanisms behind the parasitism by this organism and lineage.

10.1128/mbio.01711-22.7 FIG S6 Phylogenetic tree of *Candidatus* Paceibacteria based on 16S rRNA gene sequences. The 16S rRNA gene-based tree was constructed using the neighbor-joining method implemented in the ARB program. The operational taxonomic units (OTUs) obtained in this study are shown in bold type in the tree. Sequences that match those of the Pac_683 probe are shown in red font. Download FIG S6, JPG file, 1.4 MB. Copyright © 2022 Kuroda et al. 2022 Kuroda et al. https://creativecommons.org/licenses/by/4.0/ This content is distributed under the terms of the Creative Commons Attribution 4.0 International license.

10.1128/mbio.01711-22.8 TABLE S1 Summary of the metagenomic bins from the *Candidatus* Patescibacteria enrichment cultures observed in this study. Download Table S1, XLSX file, 0.03 MB. Copyright © 2022 Kuroda et al. 2022 Kuroda et al. https://creativecommons.org/licenses/by/4.0/ This content is distributed under the terms of the Creative Commons Attribution 4.0 International license.

10.1128/mbio.01711-22.9 TABLE S2 Summary of the recovered 16S rRNA gene sequences from culture system A-d2-33 using EMIRGE software. Download Table S2, XLSX file, 0.02 MB. Copyright © 2022 Kuroda et al. 2022 Kuroda et al. https://creativecommons.org/licenses/by/4.0/ This content is distributed under the terms of the Creative Commons Attribution 4.0 International license.

10.1128/mbio.01711-22.10 TABLE S3 Summary of the annotation of metagenomic bin PMX_810_sub using DRAM, BlastKOALA, and SignalP annotation software. Download Table S3, XLSX file, 0.2 MB. Copyright © 2022 Kuroda et al. 2022 Kuroda et al. https://creativecommons.org/licenses/by/4.0/ This content is distributed under the terms of the Creative Commons Attribution 4.0 International license.

In total, through the first successful cultivation and enrichment of the *Ca*. Patescibacteria class *Ca.* Paceibacteria (to which *Ca.* Yanofskybacteria/UBA5738 belongs), we discovered that *Ca*. Patescibacteria/CPR can symbiotically interact with the domain Archaea. The obtained results suggest that the observed interaction between *Ca.* Yanofskybacteria/UBA5738 and *Methanothrix* is a host-specific parasitism. Although the host range and the preference of *Ca.* Yanofskybacteria/UBA5738 remain unclear, the observed ability of *Ca.* Patescibacteria to interact with methanogenic archaea, a central group of organisms in anaerobic ecosystems, warrants further investigation into how parasitism may influence ecology and carbon cycling. As the presented archaea cocultivation strategy was effective in culturing *Ca*. Patescibacteria that were inhabiting methanogenic environments, we anticipate that the further refinement of cocultivation combined with gene and protein expression will allow for the characterization of the details of the symbiosis between *Ca*. Patescibacteria and Archaea, the determination of the diversity of archaea-dependent *Ca*. Patescibacteria, and, ultimately, the elucidation of the influence of these organisms’ interactions on anaerobic ecology.
