# Supplementary material for: Symbiosis between Candidatus Patescibacteria and Archaea Discovered in Wastewater-Treating Bioreactors
Source: mBio. 2022 Aug 31;13(5):e01711-22. doi: 10.1128/mbio.01711-22 (PMC9600506; doi:10.1128/mbio.01711-22)
Supplement: TEXT S1 [file mbio.01711-22-s0007.pdf]

Symbiosis between *Candidatus* Patescibacteria and Archaea discovered in wastewater-treating bioreactors

– Supporting information–

Kyohei Kuroda[1*], Kyosuke Yamamoto[1], Ryosuke Nakai[1], Yuga Hirakata[2], Kengo Kubota[3], Masaru K. Nobu[2*], and Takashi Narihiro[1*]

[1]Bioproduction Research Institute, National Institute of Advanced Industrial Science and Technology (AIST), 2-17-2-1 Tsukisamu-Higashi, Toyohira-ku, Sapporo, Hokkaido, 062-8517 Japan
[2]Bioproduction Research Institute, National Institute of Advanced Industrial Science and Technology (AIST), Central 6, Higashi 1-1-1, Tsukuba, Ibaraki 305-8566, Japan
[3]Department of Frontier Sciences for Advanced Environment, Graduate School of Environmental Studies, Tohoku University, 6-6-06 Aza-Aoba, Aramaki, Aoba-ku, Sendai, Miyagi 980-8579, Japan

[*]Co-corresponding authors:
Kyohei Kuroda, E-mail: k.kuroda@aist.go.jp
Masaru K. Nobu, E-mail: m.nobu@aist.go.jp
Takashi Narihiro, E-mail: t.narihiro@aist.go.jp

Materials and Methods

Enrichment culture experiments

Granular sludge samples were collected from a mesophilic (37°C) UASB reactor treating PET manufacturing synthetic wastewater under 94% chemical oxygen demand (COD) removal efficiency (organic loading rate: 9.4 kgCOD·m^{-3}·day^{-1}) (1) used as a microbial source for the enrichment cultures. The liquid volume of the UASB reactor was 3 L. The basal media were prepared according to previous study (2). The enrichment culture experiments were performed at 37ºC using 50 mL of serum vials containing 20 mL medium under N_2/CO_2 (80:20, v/v) atmosphere. We prepared three culture media as (A) 1m M acetate, 0.03% (w/v), 0.1 mM adenosine 5'-monophosphate, uridine 5'-monophosphate, guanosine 5'-monophosphate, and cytidine 5'-monophosphate, 1% (w/v) MEM Non-essential Amino Acids Solution (cat no. 139-15651, FUJIFILM Wako Pure Chemical Co. Ltd., Tokyo, Japan), 1% (w/v) MEM Essential Amino Acids Solution (cat no. 132-15641, FUJIFILM Wako Pure Chemical Co. Ltd., Tokyo, Japan), *Methanothrix soehngenii* GP6 (DSM 3671, 0.2 mL/20mL-medium), and *Methanosarcina barkeri* MS (DSM 800, 0.2 mL/20mL-medium), (B) A-medium without acetate, and (C) A-medium without yeast extract. *Methanothrix soehngenii* GP6 (DSM 3671) was pre-cultivated at 37ºC for 4 weeks using the 60 mM acetate and 10 mM potassium bicarbonate as substrates. *Methanosarcina barkeri* MS (DSM 800) was pre-cultivated at 37ºC for 1 week using 10 mM methanol, 0.03% yeast extract (w/v), and 10 mM acetate as substrates. These pure cultures were transferred into the enrichment culture systems as expected host of *Candidatus* Patescibacteria. For the enrichment cultures, we prepared 4th serial dilution (10^{-1}, 10^{-3}, 10^{-4}, and 10^{-6}) for each culture system. Sixteen culture systems were routinely monitored by microscope equipped with phase-contrast apparatus (BX-53, Olympus, Japan) and measurement of biogas production volumes. To further enrich the targeting microorganisms, we transferred 2 mL of 1st dilution (defined as d1) of culture system B (defined as B-d1) and 2nd dilution of culture systems A (A-d2) and 3 (C-d2) into fresh medium (defined as A-d2-d1, B-d1-d1, and C-d2-d1) on days 33 under same substrates condition as shown in above. The biogas compositions (CH_4, CO_2, N_2, and H_2) were determined by gas chromatography (Shimadzu, GC-8A, Japan) with a thermal conductivity detector fitted with a SHINCARBON-ST 50/80 stainless steel Column 4.0 m × 3.0 mm (ID) according to previous study (3).

16S rRNA gene sequence analysis

One mL of cultivated microorganisms was collected from 1st-batch of each enrichment culture system on days 12 (1st and 2nd dilution of 1st batch culture systems A–D) and 33 (only A-d2) and collected microbial cells by centrifugation at 17,750 g. DNA was extracted from microbial cells using FastDNA Spin Kit for Soil (MP Biomedicals, Santa Ana, California, USA) according to the manufacturer's protocol. The 16S rRNA genes were amplified using Univ515F–Univ909R according to a previous study (4, 5). The PCR products were purified using a QIAquick PCR purification kit (Qiagen, Valencia, CA, USA) according to the manufacturer's protocol. The sequence analysis was performed by the MiSeq Reagent kit v3 and MiSeq system (Illumina, San Diego, CA, USA). Raw 16S rRNA gene sequences were analyzed using the QIIME 2 ver. 2021.4 (6) according to previous study [2]. The 16S rRNA gene sequences were clustered by ≥ 97% similarity to operational taxonomic units (OTUs) using vsearch software (7). Taxonomic classification was carried out using the classify-sklearn retained on the SILVA database version 138 (8).

Shotgun metagenomic sequence analysis

The DNA extracted from A-d2 on cultivation days 33, and B-d1 and C-d1 on days 12 were prepared with the ThruPLEX DNA-Seq Kit (Clontech, USA) (average length: 333–363 bp). The prepared libraries were sequenced on a NovaSeq6000 (Illumina). The generated raw reads were trimmed using Trimmomatic 0.39

(SLIDINGWINDOW: 6:30 MINLEN: 100) (9). De novo assembly was performed using Megahit v1.2.9 (--k-min 27 --k-max 141 --k-step 12) (10). Co-assembly of the sequences from A-d2, B-d1, and C-d1 was performed. The assembled contigs of short length (< 2,500 bp) were removed before binning procedure. Binning was performed using Metabat2 version 2.2.7 (11), MaxBin2 version 2.15 (-markerset 40 and 107) (12), MyCC (MyCC_2017.ova) (13), and Vamb version 3.0.3 (14) with default parameters. To obtain the high-quality metagenomic bins, obtained bins from the multiple binning results were refined using Das Tool version 1.1.2 (--score_threshold 0.3 --duplicate_penalty 0.3 --megabin_penalty 0.3) (15). In the final step, the dereplication and genomic quality were checked by dRep version 3.2.0 (-comp 50 -con 10) (16). The taxonomic classification of the bins was estimated using GTDBtk v2.0.0 (GTDB release207; default parameters) (17).
The median/mean coverage of the metagenomic bins was calculated by Metabat2 pipelines (jgi_summarize_bam_contig_depths, default parameters). Relative abundance of the metagenomic bins were calculated using coverm 0.4.0 (https://github.com/wwood/CoverM). All bins were annotated through a combination of Prokka v1.14.6 (18), BlastKOALA (19), and DRAM software (--use_uniref option with default setting) (20) and manual annotation. Genomic tree was constructed using concatenated phylogenetic marker genes of obtained bin and order *Ca*. Paceibactelales genomes. Conserved marker genes were identified using "gtdbtk identify" with default parameters and aligned to reference genomes using "gtdbtk align" with taxonomic filters (--taxa_filter o__Paceibacterales) (17). Phylogenetic tree was constructed using IQ-TREE version 2.1.4-beta (-B 1000) with outomatically optimized substitution model (yeast+F+R9) (21). For the manual annotation, metagenomic bins were assigned using blastp version 2.6.0 to reference genomes of family UBA5738 and GCA-002779355. Signal peptides of the genomes were annotated using SignalP 6.0 (22). Full-length 16S rRNA gene sequence was reconstructed through EMIRGE software (-l 151 -i 148 -s 50 --phred33) (23).

Fluorescence *in situ* hybridization and phylogenetic analysis based on 16S rRNA gene sequences
On cultivation days 23, approximately 1 mL of cultivated media from 2nd-batch of enrichment culture systems A-d2-d1, B-d1-d1, and C-d2-d1 was sampled and fixed with 4% paraformaldehyde in phosphate-buffered saline (PBS) for 4 hours at 4ºC and stored in 50% ethanol with PBS at -20ºC. Fluorescence *in situ* hybridization (FISH) was performed as described previously (24). The hybridization and washing slides were 46ºC for 6–8 hours and 48ºC for 20 mins, respectively. An equimolar mixture (defined as EUB338mix) of EUB338 (5'-GCTGCCTCCCGTAGGAGT-3') (25), EUB338I (5'- GCAGCCTCCCGTAGGAGT-3'), EUB338II (5'-GCAGCCACCCGTAGGTGT-3'), and EUB338III (5'- GCTGCCACCCGTAGGTGT-3') (26) were used for detection of all bacteria. Formamide concentrations used in this study were follows: EUB338mix, 10%; ARC915 (5'- GTGCTCCCCCGCCAATTCCT-3') (27), 35%; MX825 (5'- TCGCACCGTGGCCGACACCTAGC-3') (27), 20%; and Pac_683 (5'- TCAACGGATTTCACCCCTACAC-3') (28), 25%. The PAC_683 shows perfect match for predominant order *Ca*. Yanofskybacteria OTU0011 (UBA5738) and the full length of the 16S rRNA gene sequence (100% similarity with OTU0011), and no perfect match for other detected *Ca*. Patescibacteria in all culture systems, which were confirmed by ARB software package version 7.0 with SILVA 138.1 database (29). All probes were labeled with FITC or Cy3. The FISH samples were also stained with 4',6-diamidino-2-phenylindole dihydrochloride (DAPI). The microscopic images were observed by epifluorescence microscope (BX-53, Olympus, Japan) with a color CCD camera (DP-74, Olympus, Japan). Phase-contrast, FISH, and DAPI micrographic images were uniformly processed across the entire images using Adjust color of Preview application on Mac OS 11.6.5, Photoshop CC (Adobe Creative Cloud), and ImageJ 1.53k (30). The clear fluorescence proportions of *Methanothrix* filamentous cells were measured using imaging software (cellSens, Olympus, Japan). Welch's t test calculation was performed using R software version 4.1.0. The phylogenetic tree of nearly full-length 16S rRNA gene sequences was constructed based on neighbor-joining methods in ARB

version 7.0 using SILVA138.1 database for small subunit rRNA gene sequences (29). The sequences between *E. coli* position of 110 and 1460 were used for phylogenetic tree construction.

Transmission electron microscopy

On cultivation days 40, approximately 1 mL of cultivated media from culture system A-d2 was sampled and sandwiched with the copper disks in liquid propane at -175ºC. After frozen of the samples, the liquid was freeze substituted with 2% glutaraldehyde, 1% tannic acid in ethanol, and 2% distilled water at -80ºC for 2 days. Dehydration was performed by anhydrous ethanol at 3 times for 30 mins each. Infiltration was carried out by propylene oxide at 2 times for 30 mins each, and the sample was put into a ratio of 7:3 mixtures of propylene oxide and resin for 1 hours. After volatilization of propylene oxide, the sample was transferred to a fresh 100% resin, and polymerized at 60ºC for 48 hours. Ultra-thin sections at 70 nm with a diamond knife using an ultramicrotome (Ultracut UCT, Leica, Vienna, Austria). The sections were stained with 2% uranyl acetate at room temperature for 15 mins and washed with distilled water followed by secondary-stained with Lead stain solutions (Sigma-Aldrich Co., Tokyo, Japan) at room temperature for 3 mins. The grids were observation by a transmission electron microscope (TEM) (JEM-1500Plus, JEOL Ltd., Tokyo, Japan) at 100 kV. Digital images were observed with a CCD camera (EM-14830RUBY2, JEOL Ltd., Tokyo, Japan). The cell diameters were measured from 8 and 5 single-cells attached on *Methanothrix*- and *Methanospirillum*-like cells, respectively. The cell volumes were calculated according to Van Wambeke and Bianchi (1985) (31).

Deposition of DNA sequence data

The raw sequence data and binned metagenome data were deposited into the DDBJ Sequence Read Archive database (DRA013834 and DRA014327). The 16S rRNA gene sequences were deposited in the DDBJ/EMBL/GenBank databases (LC715096–LC715128).

References

1. Kuroda K, Narihiro T, Shinshima F, Yoshida M, Yamaguchi H, Kurashita H, Nakahara N, Nobu MK, Noguchi TQP, Yamauchi M, Yamada M. 2022. High-rate cotreatment of purified terephthalate and dimethyl terephthalate manufacturing wastewater by a mesophilic upflow anaerobic sludge blanket reactor and the microbial ecology relevant to aromatic compound degradation. Water Res 219:118581.
2. Narihiro T, Nobu MK, Kim N-K, Kamagata Y, Liu W-T. 2015. The nexus of syntrophy-associated microbiota in anaerobic digestion revealed by long-term enrichment and community survey. Environ Microbiol 17:1707–1720.
3. Kuroda K, Shinshima F, Tokunaga S, Noguchi TQP, Yamauchi M, Nobu MK, Narihiro T, Yamada M. 2021. Assessing the effect of green tuff as a novel natural inorganic carrier on methane-producing activity of an anaerobic sludge microbiome. Environ Technol Innov 24:101835.
4. Kuroda K, Nobu MK, Mei R, Narihiro T, Bocher BTW, Yamaguchi T, Liu W-T. 2016. A single-granule-level approach reveals ecological heterogeneity in an upflow anaerobic sludge blanket reactor. PLoS One 11.
5. Kuroda K, Narihiro T, Shinshima F, Yoshida M, Yamaguchi H, Kurashita H, Nakahara N, Nobu MK, Noguchi TQP, Yamauchi M, Yamada M. 2022. High-rate cotreatment of purified terephthalate and dimethyl terephthalate manufacturing wastewater by a mesophilic upflow anaerobic sludge blanket reactor and the microbial ecology relevant to aromatic compound degradation. Water Res 219:118581.
6. Bolyen E, Rideout JR, Dillon MR, Bokulich NA, Abnet CC, Al-Ghalith GA, Alexander H, Alm EJ, Arumugam M, Asnicar F, Bai Y, Bisanz JE, Bittinger K, Brejnrod A, Brislawn CJ, Brown CT, Callahan

BJ, Caraballo-Rodríguez AM, Chase J, Cope EK, Da Silva R, Diener C, Dorrestein PC, Douglas GM, Durall DM, Duvallet C, Edwardson CF, Ernst M, Estaki M, Fouquier J, Gauglitz JM, Gibbons SM, Gibson DL, Gonzalez A, Gorlick K, Guo J, Hillmann B, Holmes S, Holste H, Huttenhower C, Huttley GA, Janssen S, Jarmusch AK, Jiang L, Kaehler BD, Kang K Bin, Keefe CR, Keim P, Kelley ST, Knights D, Koester I, Kosciolek T, Kreps J, Langille MGI, Lee J, Ley R, Liu Y-X, Loftfield E, Lozupone C, Maher M, Marotz C, Martin BD, McDonald D, McIver LJ, Melnik A V., Metcalf JL, Morgan SC, Morton JT, Naimey AT, Navas-Molina JA, Nothias LF, Orchanian SB, Pearson T, Peoples SL, Petras D, Preuss ML, Pruesse E, Rasmussen LB, Rivers A, Robeson MS, Rosenthal P, Segata N, Shaffer M, Shiffer A, Sinha R, Song SJ, Spear JR, Swafford AD, Thompson LR, Torres PJ, Trinh P, Tripathi A, Turnbaugh PJ, Ul-Hasan S, van der Hooft JJJ, Vargas F, Vázquez-Baeza Y, Vogtmann E, von Hippel M, Walters W, Wan Y, Wang M, Warren J, Weber KC, Williamson CHD, Willis AD, Xu ZZ, Zaneveld JR, Zhang Y, Zhu Q, Knight R, Caporaso JG. 2019. Reproducible, interactive, scalable and extensible microbiome data science using QIIME 2. Nat Biotechnol 37:852–857.

7. Rognes T, Flouri T, Nichols B, Quince C, Mahé F. 2016. VSEARCH: a versatile open source tool for metagenomics. PeerJ 4:e2584.
8. Yilmaz P, Parfrey LW, Yarza P, Gerken J, Pruesse E, Quast C, Schweer T, Peplies J, Ludwig W, Glöckner FO. 2014. The SILVA and "All-species Living Tree Project (LTP)" taxonomic frameworks. Nucleic Acids Res 42:D643–D648.
9. Bolger AM, Lohse M, Usadel B. 2014. Trimmomatic: a flexible trimmer for Illumina sequence data. Bioinformatics 30:2114–2120.
10. Li D, Liu C-M, Luo R, Sadakane K, Lam T-W. 2015. MEGAHIT: an ultra-fast single-node solution for large and complex metagenomics assembly via succinct de Bruijn graph. Bioinformatics 31:1674–1676.
11. Kang DD, Li F, Kirton E, Thomas A, Egan R, An H, Wang Z. 2019. MetaBAT 2: an adaptive binning algorithm for robust and efficient genome reconstruction from metagenome assemblies. PeerJ 7:e7359.
12. Wu Y-W, Simmons BA, Singer SW. 2016. MaxBin 2.0: an automated binning algorithm to recover genomes from multiple metagenomic datasets. Bioinformatics 32:605–607.
13. Lin H-H, Liao Y-C. 2016. Accurate binning of metagenomic contigs via automated clustering sequences using information of genomic signatures and marker genes. Sci Rep 6:24175.
14. Nissen JN, Johansen J, Allesøe RL, Sønderby CK, Armenteros JJA, Grønbech CH, Jensen LJ, Nielsen HB, Petersen TN, Winther O, Rasmussen S. 2021. Improved metagenome binning and assembly using deep variational autoencoders. Nat Biotechnol 39:555–560.
15. Sieber CMK, Probst AJ, Sharrar A, Thomas BC, Hess M, Tringe SG, Banfield JF. 2018. Recovery of genomes from metagenomes via a dereplication, aggregation and scoring strategy. Nat Microbiol 3:836–843.
16. Olm MR, Brown CT, Brooks B, Banfield JF. 2017. dRep: a tool for fast and accurate genomic comparisons that enables improved genome recovery from metagenomes through de-replication. ISME J 11:2864–2868.
17. Chaumeil P-A, Mussig AJ, Hugenholtz P, Parks DH. 2019. GTDB-Tk: a toolkit to classify genomes with the Genome Taxonomy Database. Bioinformatics 36:1925–1927.
18. Seemann T. 2014. Prokka: rapid prokaryotic genome annotation. Bioinformatics 30:2068–2069.
19. Kanehisa M, Sato Y, Morishima K. 2016. BlastKOALA and GhostKOALA: KEGG Tools for Functional Characterization of Genome and Metagenome Sequences. J Mol Biol 428:726–731.
20. Shaffer M, Borton MA, McGivern BB, Zayed AA, La Rosa SL, Solden LM, Liu P, Narrowe AB, Rodríguez-Ramos J, Bolduc B, Gazitúa MC, Daly RA, Smith GJ, Vik DR, Pope PB, Sullivan MB,

Roux S, Wrighton KC. 2020. DRAM for distilling microbial metabolism to automate the curation of microbiome function. Nucleic Acids Res 48:8883–8900.

21. Minh BQ, Schmidt HA, Chernomor O, Schrempf D, Woodhams MD, Von Haeseler A, Lanfear R, Teeling E. 2020. IQ-TREE 2: New Models and Efficient Methods for Phylogenetic Inference in the Genomic Era. Mol Biol Evol 37:1530–1534.
22. Teufel F, Almagro Armenteros JJ, Johansen AR, Gíslason MH, Pihl SI, Tsirigos KD, Winther O, Brunak S, von Heijne G, Nielsen H. 2022. SignalP 6.0 predicts all five types of signal peptides using protein language models. Nat Biotechnol 40:1023–1025.
23. Miller CS, Baker BJ, Thomas BC, Singer SW, Banfield JF. 2011. EMIRGE: reconstruction of full-length ribosomal genes from microbial community short read sequencing data. Genome Biol 12:R44.
24. Sekiguchi Y, Kamagata Y, Nakamura K, Ohashi A, Harada H. 1999. Fluorescence In Situ Hybridization Using 16S rRNA-Targeted Oligonucleotides Reveals Localization of Methanogens and Selected Uncultured Bacteria in Mesophilic and Thermophilic Sludge Granules. Appl Environ Microbiol 65:1280–1288.
25. Amann RI, Binder BJ, Olson RJ, Chisholm SW, Devereux R, Stahl DA. 1990. Combination of 16S rRNA-targeted oligonucleotide probes with flow cytometry for analyzing mixed microbial populations. Appl Environ Microbiol 56:1919–1925.
26. Daims H, Brühl A, Amann R, Schleifer K-H, Wagner M. 1999. The Domain-specific Probe EUB338 is Insufficient for the Detection of all Bacteria: Development and Evaluation of a more Comprehensive Probe Set. Syst Appl Microbiol 22:434–444.
27. Raskin L, Stromley JM, Rittmann BE, Stahl D a. 1994. Group-specific 16S rRNA hybridization probes to describe natural communities of methanogens. Appl Environ Microbiol 60:1232–1240.
28. Singleton CM, Petriglieri F, Kristensen JM, Kirkegaard RH, Michaelsen TY, Andersen MH, Kondrotaite Z, Karst SM, Dueholm MS, Nielsen PH, Albertsen M. 2021. Connecting structure to function with the recovery of over 1000 high-quality metagenome-assembled genomes from activated sludge using long-read sequencing. Nat Commun 12:2009.
29. Ludwig W, Strunk O, Westram R, Richter L, Meier H, Yadhukumar, Buchner A, Lai T, Steppi S, Jobb G, Förster W, Brettske I, Gerber S, Ginhart AW, Gross O, Grumann S, Hermann S, Jost R, König A, Liss T, Lüssmann R, May M, Nonhoff B, Reichel B, Strehlow R, Stamatakis A, Stuckmann N, Vilbig A, Lenke M, Ludwig T, Bode A, Schleifer K-H. 2004. ARB: a software environment for sequence data. Nucleic Acids Res 32:1363–71.
30. Schneider CA, Rasband WS, Eliceiri KW. 2012. NIH Image to ImageJ: 25 years of image analysis. Nat Methods 9:671–675.
31. Van Wambeke F, Bianchi M. 1985. Bacterial biomass production and ammonium regeneration in Mediterranean sea water supplemented with amino acids. 2. Nitrogen flux through heterotrophic microplankton food chain. Mar Ecol Prog Ser 23:117–128.
