# Supplementary figures and images for: Symbiosis between Candidatus Patescibacteria and Archaea Discovered in Wastewater-Treating Bioreactors
Source: mBio. 2022 Aug 31;13(5):e01711-22. doi: 10.1128/mbio.01711-22 (PMC9600506; doi:10.1128/mbio.01711-22)

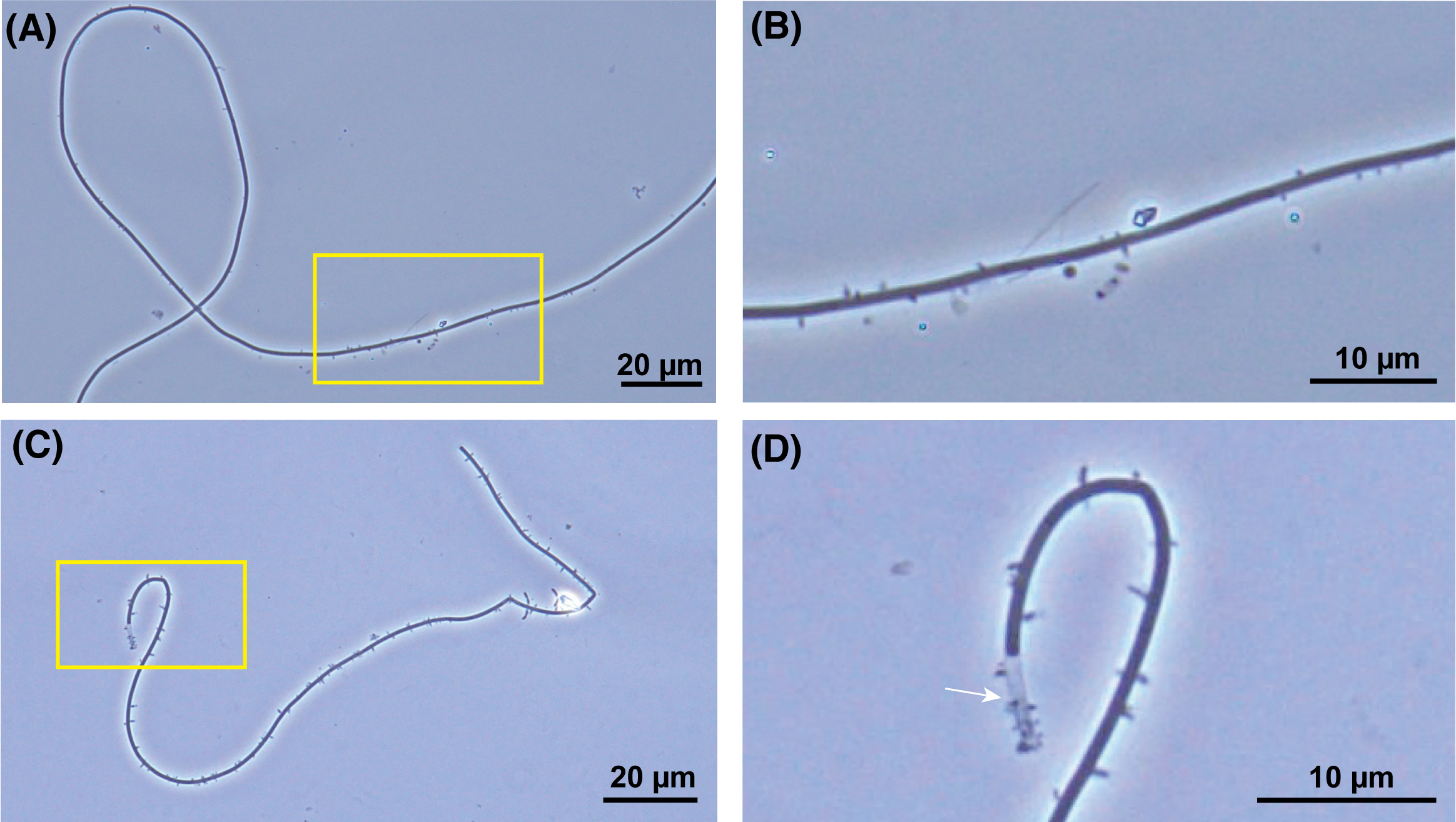

Supplement: FIG S1 [file mbio.01711-22-s0001.jpg]

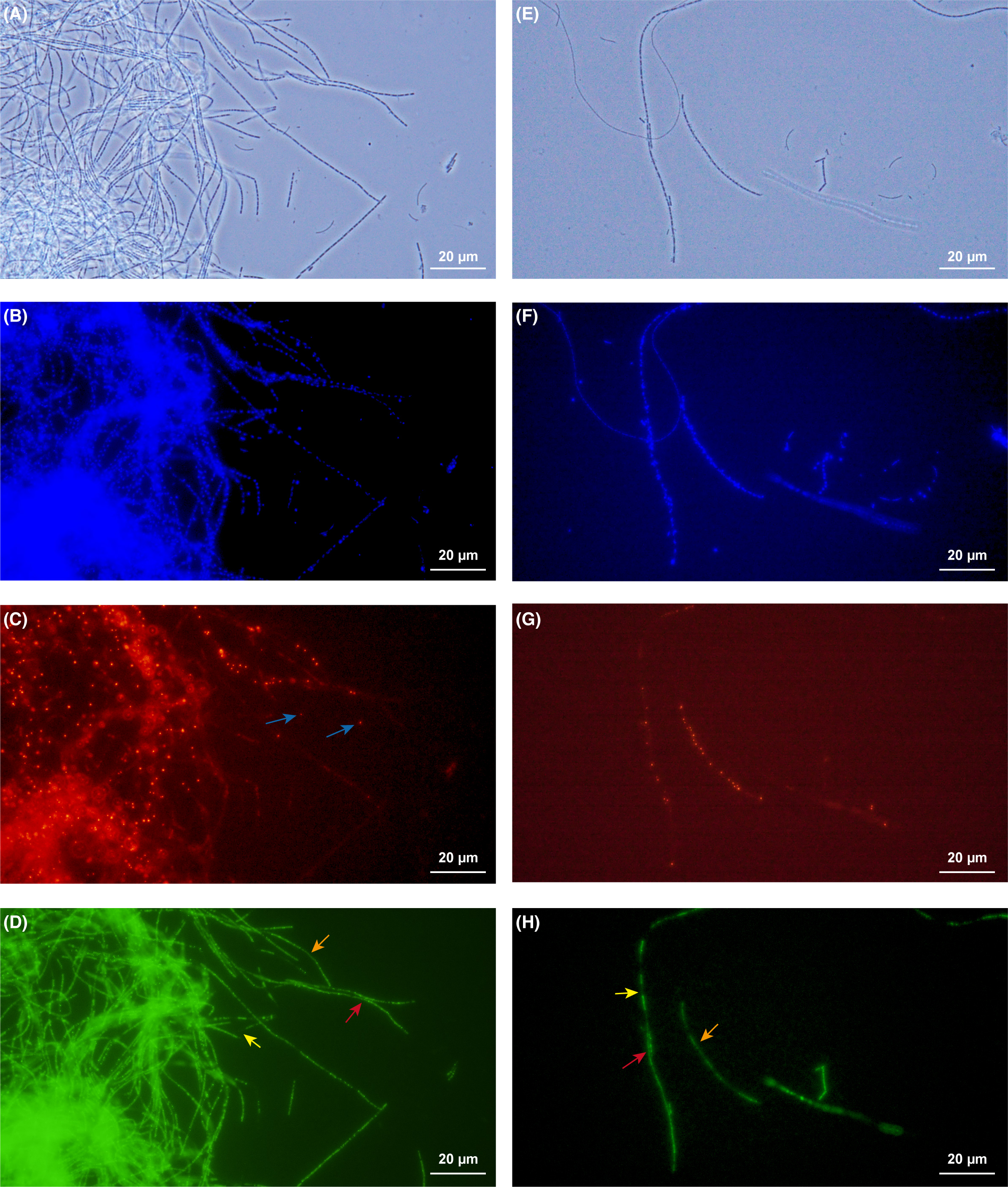

Supplement: FIG S2 [file mbio.01711-22-s0002.jpg]

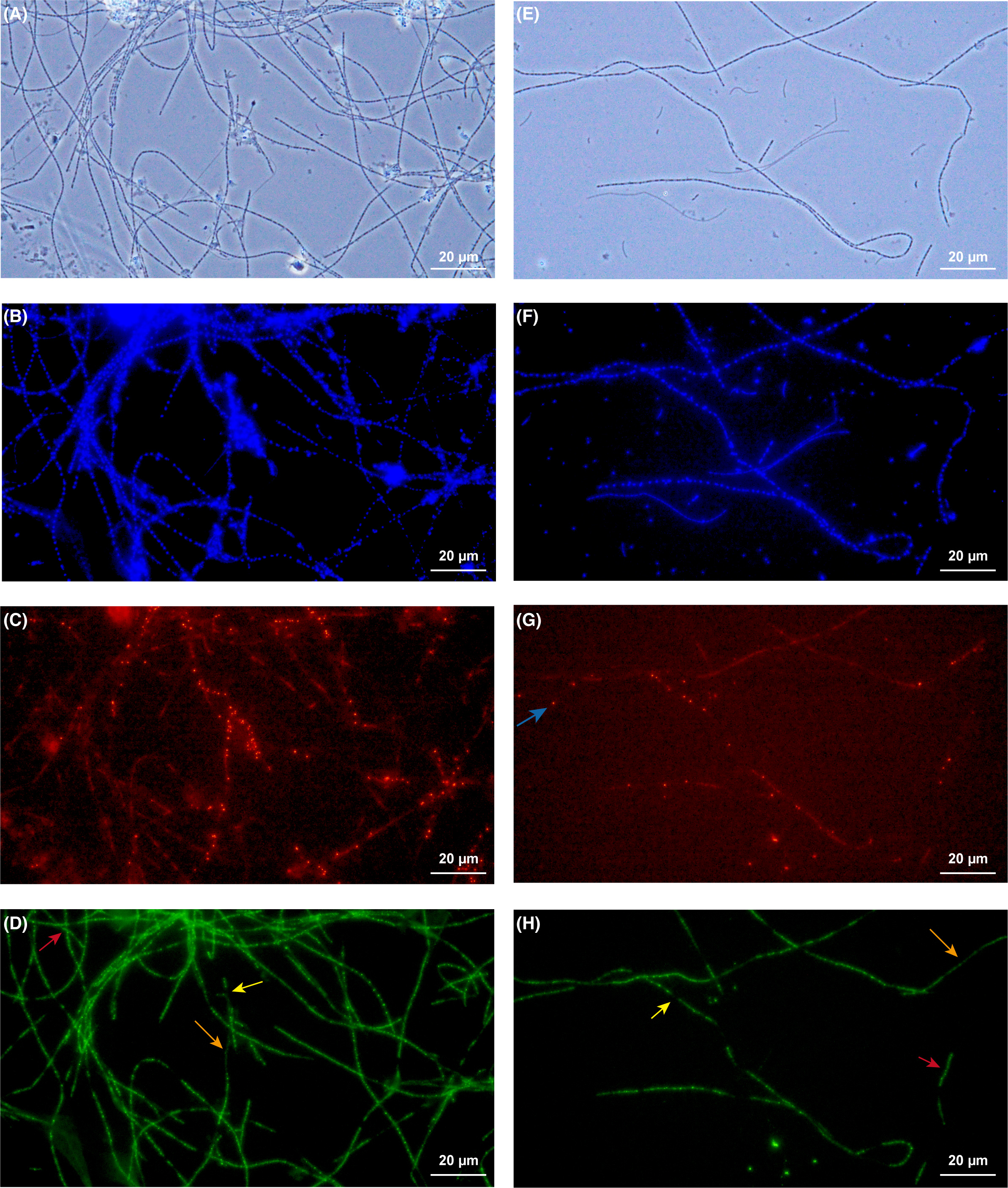

Supplement: FIG S3 [file mbio.01711-22-s0003.jpg]

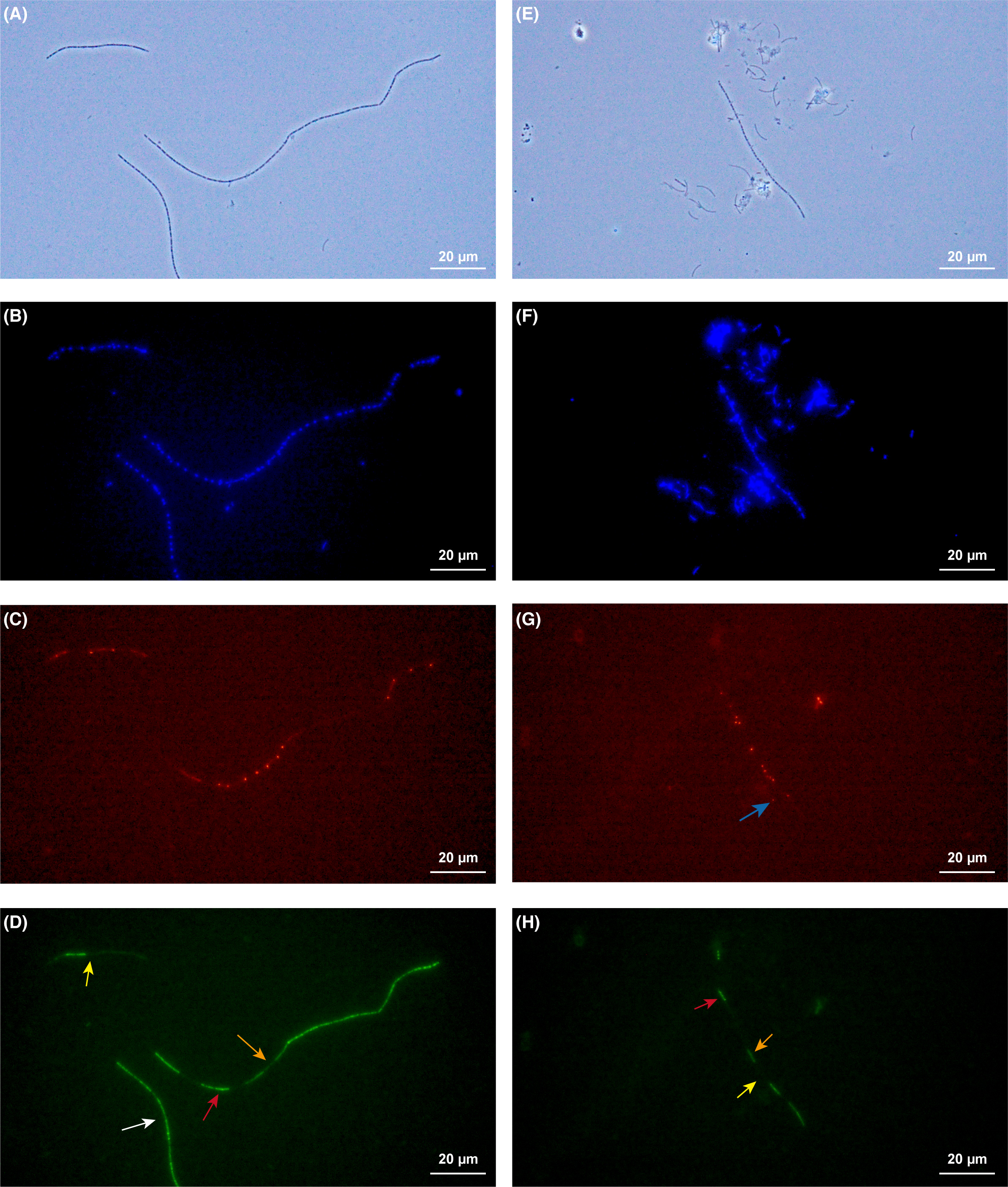

Supplement: FIG S4 [file mbio.01711-22-s0004.jpg]

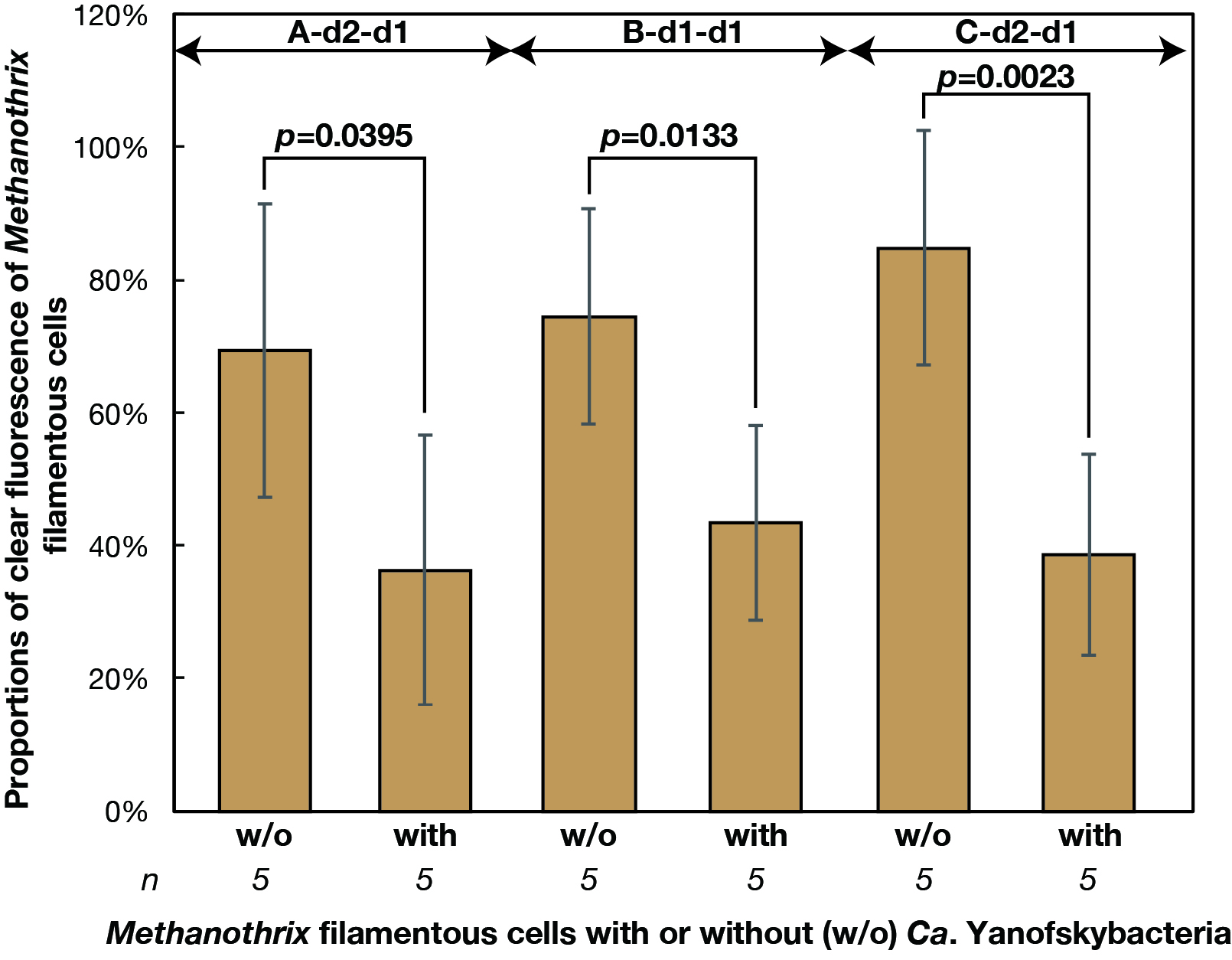

Supplement: FIG S5 [file mbio.01711-22-s0005.jpg]

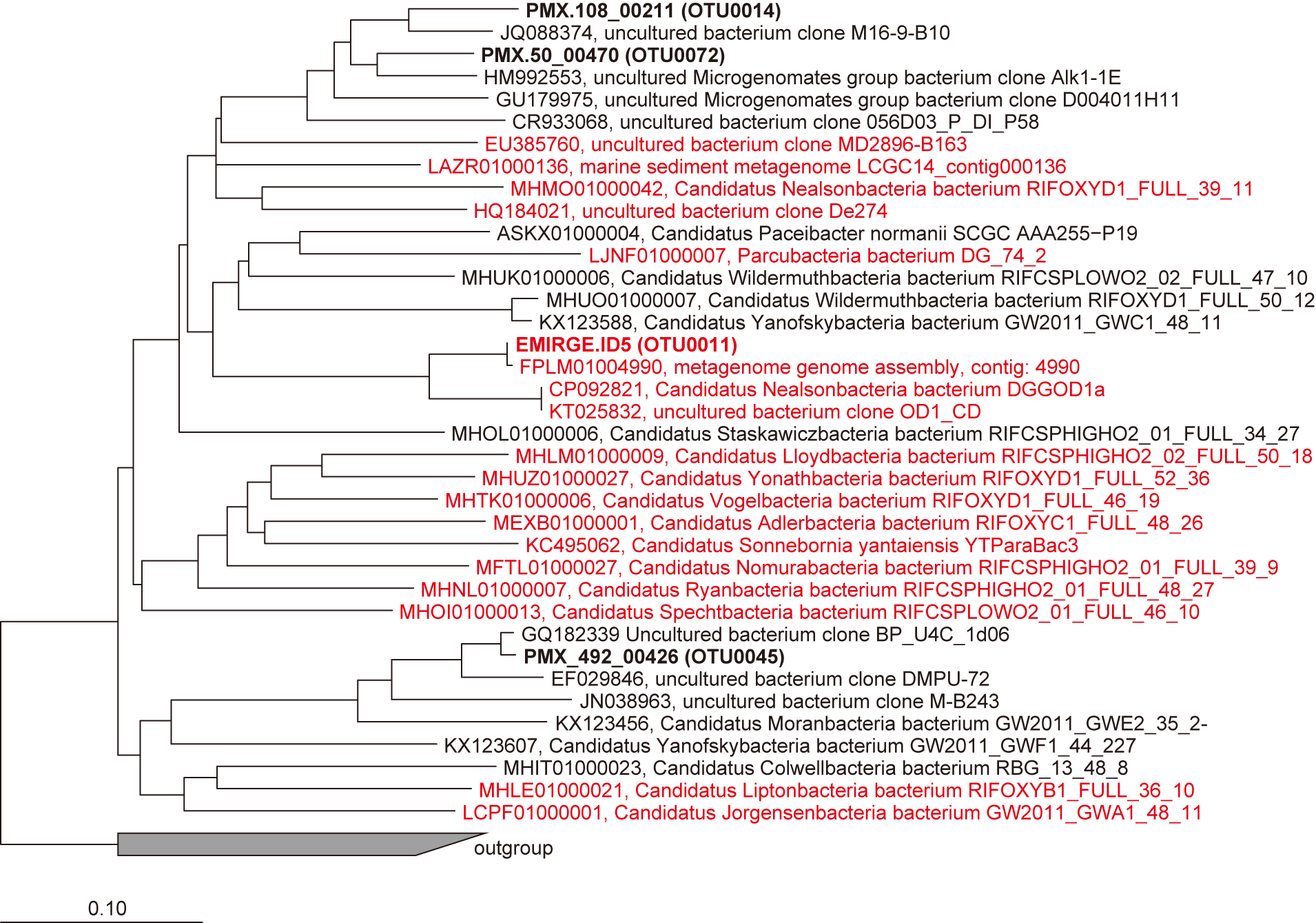

Supplement: FIG S6 [file mbio.01711-22-s0006.jpg]
